# Adaptive Noise Reduction of Scintigrams with a Wavelet Transform

**DOI:** 10.1155/2012/130482

**Published:** 2012-02-28

**Authors:** Koichi Ogawa, Masahiko Sakata, Yu Li

**Affiliations:** Department of Applied Informatics, Faculty of Science and Engineering, Hosei University, Tokyo 184-8584, Japan

## Abstract

The aim of this study was to eliminate the effect of Poisson noise in scintigrams with a wavelet thresholding method. We developed a new noise reduction method with a wavelet transform. The proposed method was a combination of the translation-invariant denoising method and our newly introduced denoising filter which was applicable for Poisson noise. To evaluate the validity of our proposed method, phantom images and scintigrams were used. The results with the phantom images showed that our method was better than conventional methods in terms of the peak signal-to-noise ratio by 3 dB. Quality of the scintigrams processed with our method was better than that with the conventional methods in terms of reducing Poisson noise while preserving edge components. The results demonstrated that the proposed method was effective for the reduction of Poisson noise in scintigrams.

## 1. Introduction

The quality of a scintigram depends on the number of acquired photons per unit area. Generally, the geometric efficiency of a gamma camera is small, and the data acquisition time of a scintigram is limited, and so the acquired counts of gamma rays are sometimes several tens to a hundred per pixel for imaging. As a result, the image is distorted with Poisson noise, making it hard to detect small uptake on a scintigram.

Many methods have been proposed to remove this noise and restore the spatial resolution in a scintigram, for example, linear filters and order statistic filters such as a median filter in the spatial domain, and Butterworth filter and Wiener filter in the frequency domain [1–11]. However, these filters sometimes reduce the edge information in the process of denoising. On the other hand, a wavelet transform [12] is useful to remove the noise selectively without loosing the edge information, and so several methods with a discrete wavelet transform have been proposed [13–20]. Donoho proposed a method called VisuShrink in reference to the good visual quality of reconstruction obtained by the simple “shrinkage” of wavelet coefficients [13]. Here, “shrinkage” means a thresholding operation. To improve the performance of denoising with the criterion of mean-squared error (MSE), SureShrink [15] has been developed to suppress noise with a threshold determined by the principle of minimizing Stein's unbiased risk estimator (SURE) [21]. The method with the concept of SURE has an advantage in that the statistics of the original image need not be considered, and SURE-LET (linear expansion of threshold) was developed [16, 17], in which the denoising was performed with a thresholding function instead of a simple threshold value. As an alternative threshold selection method, BayesShrink [18] was developed to reduce the noise adaptively, in which a different threshold derived in a Bayesian framework is applied to the wavelet coefficients for each pixel. This method is more effective in removing Gaussian noise than SureShrink, but is insufficient to remove the Poisson noise appearing in a scintigram. The noise reduction methods with a wavelet transform including the above methods commonly assume the uniform distribution of Gaussian noise on an image and so do not always prove effective for the Poisson noise whose variance is equivalent to the mean value of the signal. On the other hand, methods to reduce Poisson noise have also been proposed by many researchers [19, 20, 22–27], and these methods sometimes work well. Of these methods, Wang's method [19, 20] with a wavelet transform can reduce the Poisson noise effectively; however, the parameter selection in this method is inappropriate for scintigrams and sometimes looses edge information in the process of denoising.

In this paper, we proposed a new method to yield high-quality scintigrams by reducing Poisson noise adaptively. In our method, we multiplied the threshold value determined with the BayesShrink method by a factor in considering the local average count around the pixel of interest and used it to reduce the Poisson noise with a filtering function. And we also applied the idea of a translation-invariant (TI) denoising [28] to reduce the artifacts caused in the process of wavelet shrinkage. In this study, we evaluated the performance of our proposed method in comparison with the conventional denoising methods

## 2. Materials and Methods

### 2.1. Proposed Method

**Figure 1:**
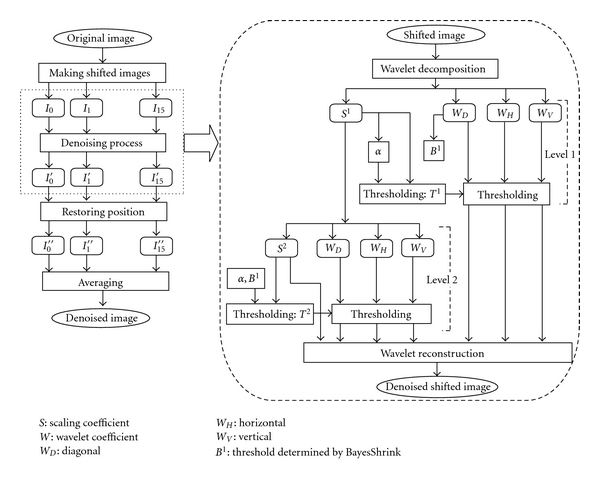
Flowchart of the proposed method. In this figure, the decomposition was performed up to level 2, but actually we decomposed to level 3.

Our proposed method consisted of the following steps.  Figure 1 shows the flowchart of our method.

**(1) Making Shifted Images**

Here, we assumed an original image *I*_0_ composed of *N* × *N* pixels, where *N* = 2^*M*^, and *M* was an integer value. To apply the concept of the TI denoising method, we made several images by shifting the pixel position in the direction of the *x*- and/or *y*-axis circularly. In this study, we used a wavelet kernel consisting of 4 taps, and so the number of images shifted here became 16 (*I*_0_ − *I*_15_), equal to 4 × 4.

**(2) Wavelet Decomposition of the 16 Shifted Images**

In the discrete wavelet transform, we used the Daubechies kernel [29, 30] and decomposed the 16 images up to level 3.

**(3) Reduction of Poisson Noise**

We applied the following method to coefficients of the above 16 images (*I*_0_ − *I*_15_) in the wavelet domain. The fluctuations of counts in a scintigram obey the Poisson distribution, and its variance differs locally depending on the number of detected photons. In the Poisson distribution, the variance equals the expected value of detected photons, and so we used scaling coefficients *S*_*xy*_^*j*^ as a reference in determining a local threshold *T*_*xy*_^*j*^ at a given position (*x*, *y*):

(1) $$T_{xy}^{j}=\frac{\alpha \times S_{xy}^{j}}{2^{j}}\cdot B^{j},$$

where *j* was a subband level, and *B* ^*j*^ was a threshold at level *j* determined by the BayesShrink method [18]. The weight *α* was determined by the scaling coefficients *S*_*xy*_^1^ at level one as follows:

(2) $$\alpha =\frac{1}{N^{2}}\frac{\sum_{y=1}^{N/2}\sum_{x=1}^{N/2}\sqrt{\left|S_{xy}^{1}\right|}}{\sum_{y=1}^{N/2}\sum_{x=1}^{N/2}\left|S_{xy}^{1}\right|}.$$

By using this weight, we took into account the local signal-to-noise ratio in (1), because the scaling coefficient is proportional to the local average of the numbers of gamma rays. The denominator is a normalizing factor.

**(4) Thresholding Function**

In our method, we used the following filtering function to remove Poisson noise while preserving the edge information:

(3) $${\tilde{\eta }}_{xy}=\{\begin{array}{cc} \mathrm{sgn}\left(\eta_{xy}\right)\left(\left|\eta_{xy}\right|-\frac{T_{xy}^{j}\exp\left(t_{0}-\left|\eta_{xy}\right|\right)}{1+\exp\left(t_{0}-\left|\eta_{xy}\right|\right)}\right) & \text{if}\;\left|\eta_{xy}\right|>T_{xy}^{j},\\ 0, & \text{otherwise}, \end{array}$$

where *η*_*xy*_ is an original wavelet coefficient, ${\tilde{\eta }}_{xy}$ is the wavelet coefficient after thresholding, and *t*_0_ is an arbitrary value that is the inflection point of (3). In this paper, we selected *t*_0_ value so as to match a value above 2% of the number of absolute wavelet coefficients from the largest value.

The filtering function works like a soft thresholding method where the absolute of the coefficients is near zero. On the other hand, it works like a hard thresholding method where the absolute of wavelet coefficients is large. And so the denoised wavelet coefficients gradually change around the threshold, and an abrupt truncation effect that appears in a hard thresholding method is avoided.

**(5) Reconstruction of the 16 Denoised Images and Averaging**

After the wavelet reconstruction of the 16 images, we restored the pixel positions to their original ones and averaged the pixel value of these 16 images pixel by pixel.

### 2.2. Phantom Data

To evaluate our proposed method quantitatively, we used two images and compared our method with conventional methods: BayesShrink [18], SURE-LET [17], and Wang's method [19]. BayesShrink estimates the variance of the Gaussian noise with a robust median estimator and applies its weighted values to each subband. SURE-LET uses SURE in determining a threshold for denoising. Wang's method uses an optimum weight that is multiplied to the threshold determined in the BayesShrink method.

**Figure 2:**
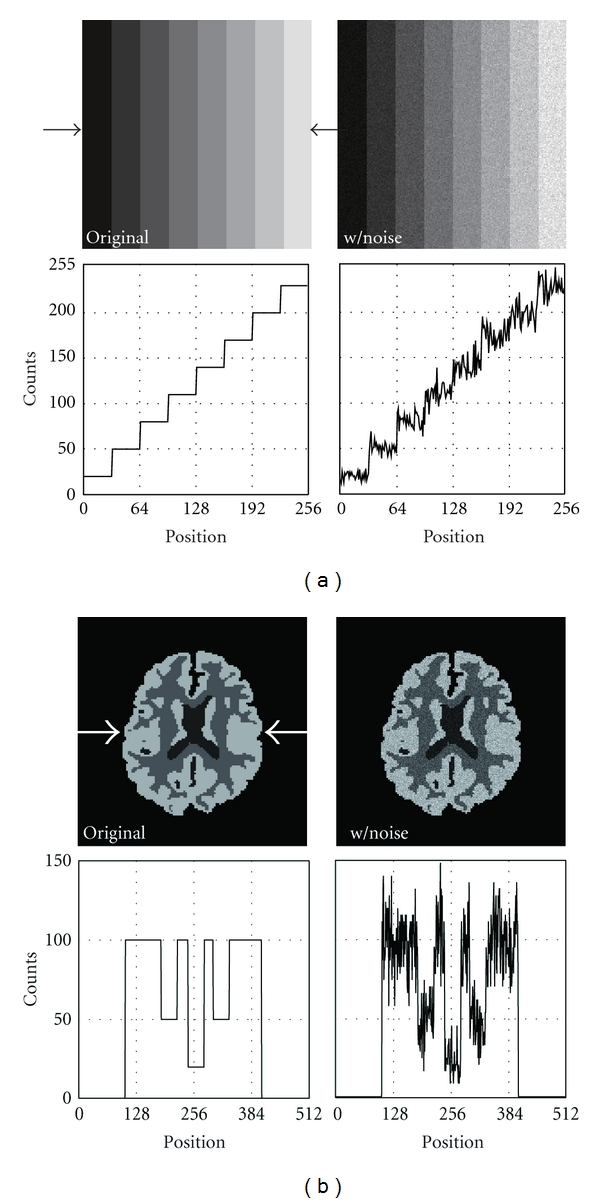
Original phantom images and those with Poisson noise. (a) Staircase phantom, (b) brain phantom. The profile along a line indicated with two arrows is shown below each image.

In the simulation, we used an image whose gray levels changed from 20 to 230 like a staircase as shown in Figure 2(a). The size of the image was 256 × 256. In addition, we used a brain image (Figure 2(b)) with a size of 512 × 512. We set three expected values (20, 50, 100) inside the brain image. Knuth's method [31] was used to add Poisson noise to the above original images. The original images with Poisson noise are shown in Figures 2(a) and 2(b). We used the Daubechies kernel (tap: 4) in the discrete wavelet transform, and the decomposition level was three. To evaluate the quality of the denoised images, we used a PSNR (peak signal-to-noise ratio) as follows:

(4) $$\begin{array}{c} \text{MSE}=\frac{1}{N^{2}}\overset{N}{\underset{x=1}{\sum }}\;\overset{N}{\underset{y=1}{\sum }}{\left(f\left(x,y\right)-g\left(x,y\right)\right)}^{2},\\ \text{PSNR}=10\;\log_{10}\left(\frac{D_{\max}^{2}}{\text{MSE}}\right), \end{array}$$

where *D*_max⁡_ means the maximum pixel value in an image, *f*(*x*, *y*) is an original image without noise, and *g*(*x*, *y*) is a denoised image. *N* × *N* is the image size in pixels. In this simulation, we used 8-bit images, and so we set *D*_max⁡_ = 255.

### 2.3. Clinical Data

To confirm the validity of our proposed method, we acquired scintigrams (^99m^Tc-MDP bone-scan and ^67^Ga-scan) with a gamma camera (GCA9300, Toshiba Medical Systems, Japan). In ^99m^Tc-scan, we used a low-energy high-resolution collimator, and in ^67^Ga-scan a medium-energy high-resolution collimator. The dose administered was 555 MBq for ^99m^Tc-scan and 111 MBq for ^67^Ga-scan, and a whole body data acquisition mode was used with a moving speed of 100 mm/min. The sizes of an image were 512 × 512 (^99m^Tc-scan) and 256 × 256 (^67^Ga-scan), and the pixel depth was 16 bits. Each image was processed with BayesShrink, SURE-LET, Wang's methods, and our proposed method. Wavelet decomposition with Daubechies kernel (tap: 4) was performed up to the third level to make a fair comparison of these methods.

## 3. Results

### 3.1. Phantom Data

**Figure 3:**
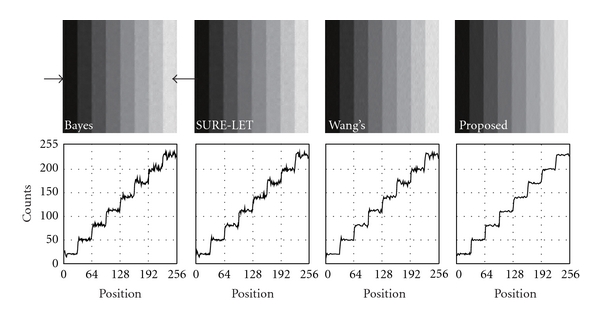
Denoised image of the staircase phantom. From left to right, BayesShrink, SURE-LET, Wang's method and our proposed method. The profile along a line indicated with two arrows is shown below each image.

**Figure 4:**
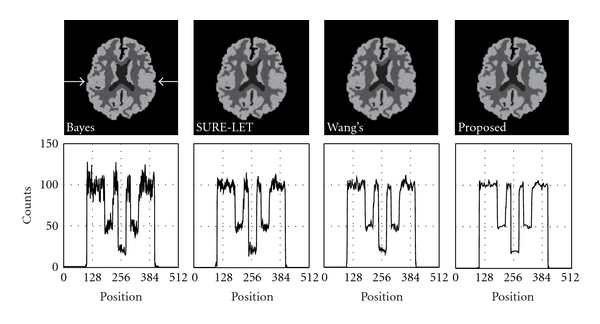
Denoised image of the brain phantom. From left to right, BayesShrink, SURE-LET, Wang's method, and our proposed method. The profile along a line indicated with two arrows is shown below each image.

The results of the staircase phantom are shown in Figure 3. In it, the images denoised with BayesShrink, SURE-LET, Wang's methods, and our proposed method are shown. To evaluate the fluctuation of pixel values quantitatively, we showed a profile of pixel values along a line indicated with two arrows.  Figure 4 shows the results of the brain phantom. We also showed the profile of pixel values in this figure. The results of the numerical evaluation with the PSNR are shown in Table 1.

### 3.2. Clinical Data

**Figure 5:**
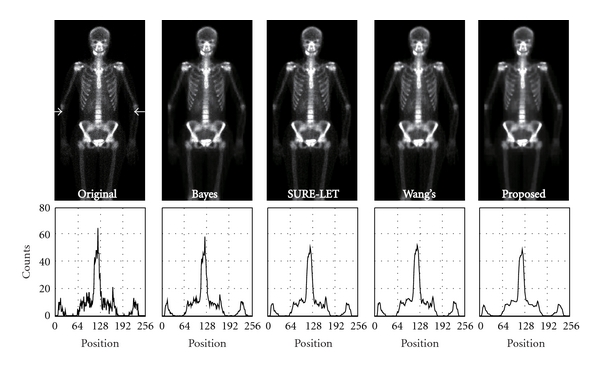
Results of the simulations with a ^99m^Tc-scintigram. From left to right: an original scintigram, BayesShrink, SURE-LET, Wang's method, and our proposed method. The profile along a line indicated with two arrows is shown below each image.

**Figure 6:**
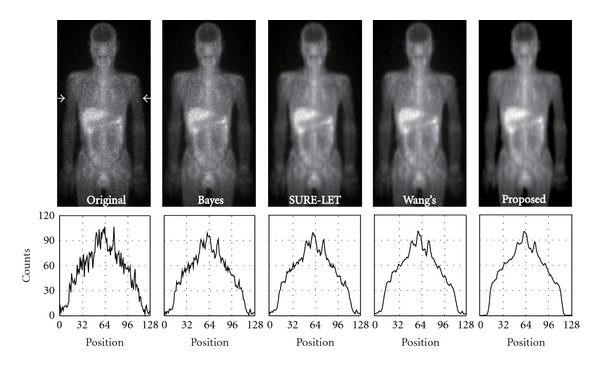
Results of the simulations with a ^67^Ga-scintigram. From left to right: an original scintigram, BayesShrink, SURE-LET, Wang's method, and our proposed method. The profile along a line indicated with two arrows is shown below each image.

The original image used in this evaluation and image denoised with BayesShrink, SURE-LET, Wang's methods, and our proposed method are shown in Figures 5 and 6.  Figure 5 shows the results of ^99m^Tc-MDP bone-scan image, and Figure 6 shows those of ^67^Ga-scan image. In these figures, only half of a processed image is shown (512 × 256 or 256 × 128). The count profile along a line indicated with two arrows is shown below each image.

## 4. Discussion

We developed a new noise reduction method for scintigrams with a wavelet transform. The main feature of our method is that the threshold value used in the wavelet shrinkage is scaled adaptively with the local average of acquired counts. Most of the early denoising methods with a wavelet transform use a fixed threshold to remove noise, and the threshold is determined with the variance of wavelet coefficients. On the other hand, BayesShrink is an effective method that changes the threshold at each level. And if we compare the results with those of linear filters in the spatial domain or the frequency domain, the BayesShrink method works well in eliminating Gaussian noise. In this method, the threshold value is determined referring to the wavelet coefficients of the diagonal components in level one, and the median of the absolute value of the wavelet coefficients is adopted as a reference value. However, if the fluctuation of the wavelet coefficients differs locally such as in the case of Poisson noise, the denoising sometimes failed as shown in Figures 2 and 3. The method works effectively when the Gaussian noise with a specified variance is distributed uniformly on an image. The application of a thresholding filter in SURE-LET yields good results by using the SURE. The method could remove the Gaussian noise almost perfectly, provided that its variance was known or correctly estimated. However, in the case of a scintigram, the denoising failed in some regions as shown in Figures 2 and 3. On the other hand, Wang's method modified the threshold determined by the BayesShrink method by multiplying a weighting factor. This approach is somewhat similar to that of our method, and the performance of denoising is better than that of the former two methods as shown in Figures 2 and 3. The major difference between our method and Wang's method is that our method refers to a local mean in the determination of a threshold of interest. The scaling coefficient is equivalent to the local mean of acquired counts, as a result of which the threshold value becomes more adaptive as compared to the other conventional methods. Figures 2 and 3 show that our method can remove Poisson noise at any count level. The performance of denoising was demonstrated numerically in Table 1. The results of the quantitative analysis with the PSNR showed that our method, which modified the threshold function slightly pixel by pixel according to the local average count, was better than the conventional methods by more than 3-dB.

 Denoising methods with a wavelet transform basically use either a soft thresholding method or hard thresholding method. The soft thresholding method reduces the amount of coefficients outside the shrinkage region, as a result of which the contrast resolution of the denoised image is decreased. On the other hand, the hard thresholding method keeps the wavelet coefficient outside the shrinkage region, and so there becomes an abrupt change in wavelet coefficients that occurs around the threshold value. This introduces ripples near sharp edges in denoised images. With regard to these artifacts that occur in the process of denoising with a shrinkage method, Coifman and Donoho proposed a method called TI denoising [28], which efficiently suppresses the artifacts due to the lack of translation invariance of the wavelet basis. In our method, we applied the concept of the TI method and successfully suppressed the artifacts appeared at the edge of a region.

 In scintigrams, there is a region outside the human body where the number of detected photons is nearly zero. This area affects the estimation of an optimal threshold, and thus, we eliminated the pixels whose values were less than 10 percent of the maximum counts in an image. With this process, we could eliminate the effect of this region and reduce the Poisson noise appropriately. As for the decomposition level, we decomposed an original image with the third level in the discrete wavelet transform. If we increase the level, the denoised images are considerably smoothed, and if we decrease the level, denoising is insufficient, and so we set the decomposition level at three. Our proposed method has only one parameter to control the shape of a filtering function. We can modify the amount of wavelet coefficients near the threshold with this parameter more adaptively depending on image features, if necessary.

## 5. Conclusion

We proposed a new method for reducing Poisson noise in scintigrams. Our method could remove Poisson noise efficiently, and a translation-invariant denoising operation suppresses artifacts occurring near the edge. The results of the simulations showed that our proposed method was better than the conventional methods by more than 3-dB in PSNR, and processed scintigrams were improved in quality without excess smoothing. We confirmed that our method was effective in reducing Poisson noise while preserving the fine structures on scintigrams.

## Figures and Tables

**Table 1:** PSNR of the phantom images in dB.

	Original with noise	BayesShrink	SURE-LET	Wang's method	Proposed method
Staircase phantom	28.17	35.84	36.37	37.44	40.75
Brain phantom	28.10	33.02	33.78	34.49	37.52
